# P239: Salmonella group B 4,5 outbreak on a neonatology unit

**DOI:** 10.1186/2047-2994-2-S1-P239

**Published:** 2013-06-20

**Authors:** M Grande, P Rodríguez, M Terol, M Triviño, C Suárez, C Gil

**Affiliations:** 1 Preventive Medicine and Quality Management, General University Hospital Gregorio Marañón. Madrid. Spain

## Introduction

We report an outbreak of *Salmonella* group B 4,5 infections in patients on a Neonatology unit, and describe the epidemiological investigations and control measures undertaken.

## Methods

After identification of various *Salmonella* group B 4,5 positive cultures from different patients staying in the Neonatology Unit, active surveillance through clinical and microbiological results was initiated.

Stool cultures were taken from all patients admitted to the unit and screening of medical staff for healthy carriers was performed.

We studied the compliance of specific precautions based on contact transmission among the personnel working in the unit but also among consulted professionals from other departments.

## Results

After the vertical transmission of *Salmonella* group B 4,5 in two twins born in the hospital in May 2012, both admitted to the neonatology unit with a diagnosis of sepsis, during the month of June four more patients were identified with *Salmonella* group B 4,5-positive cultures, of whom one presented sepsis, one suffered from gastroenteritis and two patients remained asymptomatic. In the following day all six patients were discharged, four of them still colonized with *Salmonella* group B4,5.

In June 2012 all stool cultures of patients admitted to the Neonatology Unit were negative. No carriers were detected among healthcare staff.

Hand hygiene compliance in the unit was 76,8%.

Possible areas of improvement were found to be hand hygiene in consulted professionals from other departments as well as the cleaning of machines and devices (monitors, ultrasound machine) after using them.

## Conclusion

Data suggested that the source of the infection was the index patient's mother who was colonized with *Salmonella* group B 4,5; the mode of transmission was most likely due to the transfer of organisms from infant to infant by cross transmission.

## Disclosure of interest

None declared

